# Sequential Isotopic Signature Along Gladius Highlights Contrasted Individual Foraging Strategies of Jumbo Squid (*Dosidicus gigas*)

**DOI:** 10.1371/journal.pone.0022194

**Published:** 2011-07-14

**Authors:** Anne Lorrain, Juan Argüelles, Ana Alegre, Arnaud Bertrand, Jean-Marie Munaron, Pierre Richard, Yves Cherel

**Affiliations:** 1 IRD, LEMAR UMR CNRS/UBO/IRD 6539, Plouzané, France; 2 IMARPE, Callao, Lima, Perú; 3 IRD, UMR212 EME IFREMER/IRD/UM2, Sète, France; 4 Littoral, Environnement et Sociétés, UMR 6250 CNRS-Université de La Rochelle, La Rochelle, France; 5 Centre d'Etudes Biologiques de Chizé, UPR 1934 du CNRS, Villers-en-Bois, France; Dalhousie University, Canada

## Abstract

**Background:**

Cephalopods play a major role in marine ecosystems, but knowledge of their feeding ecology is limited. In particular, intra- and inter-individual variations in their use of resources has not been adequatly explored, although there is growing evidence that individual organisms can vary considerably in the way they use their habitats and resources.

**Methodology/Principal Findings:**

Using δ^13^C and δ^15^N values of serially sampled gladius (an archival tissue), we examined high resolution variations in the trophic niche of five large (>60 cm mantle length) jumbo squids (*Dosidicus gigas*) that were collected off the coast of Peru. We report the first evidence of large inter-individual differences in jumbo squid foraging strategies with no systematic increase of trophic level with size. Overall, gladius δ^13^C values indicated one or several migrations through the squid's lifetime (∼8–9 months), during which δ^15^N values also fluctuated (range: 1 to 5‰). One individual showed an unexpected terminal 4.6‰ δ^15^N decrease (more than one trophic level), thus indicating a shift from higher- to lower-trophic level prey at that time. The data illustrate the high diversity of prey types and foraging histories of this species at the individual level.

**Conclusions/Significance:**

The isotopic signature of gladii proved to be a powerful tool to depict high resolution and ontogenic variations in individual foraging strategies of squids, thus complementing traditional information offered by stomach content analysis and stable isotopes on metabolically active tissues. The observed differences in life history strategies highlight the high degree of plasticity of the jumbo squid and its high potential to adapt to environmental changes.

## Introduction

Pelagic squids play a major role in the trophic structure of marine ecosystems as they constitute large pelagic biomasses, and are voracious predators and important prey for other organisms [1]. However, knowledge of their life history traits, including feeding strategies and diets is limited by biases resulting from net feeding and subsequent stomach content analyses [2]. Individual variation (intra- and inter-) in their use of resources is not well known, although there is growing evidence that individuals in a population vary considerably in the way they use their habitats and resources [3], [4].

A new tool to investigate long-term trophic ecology at both the population and individual levels has been recently developed by combining stable isotope measurements and sequential sampling of hard tissues that remain inert after synthesis [5]–[8]. Carbon isotopes (δ^13^C) can be used to investigate the animals' habitats and migration patterns, while δ^15^N values depict the associated trophic variations [5]. Indeed, in contrast to δ^15^N values which show a stepwise enrichment from prey to predators [9], carbon isotope values vary little with each trophic level. They therefore mostly reflect spatial variations of the environment and can indicate offshore vs. inshore, benthic vs. pelagic feeding, or even latitudinal variations in foraging habitats [10], [11]. In squids, stable isotope profiles along the gladius, their internal chitinous shell, have been recently analyzed and seem to be a promising tool to produce a chronological record of dietary information over their lifetime [12], [13]. The gladius grows continuously by accretion of new molecules of chitin and proteins at the proximal part of the gladius (near the head), with no metabolic turnover after synthesis. Consequently, it retains molecules sequentially laid down throughout the lives of cephalopods, and sequential sampling along this structure gives a dietary record of the organism throughout its life.

The jumbo squid (*Dosidicus gigas*, Ommastrephidae) has a strong economic and ecological importance in the eastern Pacific Ocean. It is a fast growing species, from 1 mm mantle length (ML) at birth to more than 1 m ML when 1–2 years old [14]. In the last decade, jumbo squids have shown a significant increase in abundance and distribution in the Eastern Pacific [15], [16]. Both climatic and anthropogenic factors impact jumbo squids and marine organisms, but a current hypothesis is that individual feeding histories of jumbo squid strongly control the population structure [17]. Here, we measured sequential isotopic values along the gladius of five large individual jumbo squids that were sampled the same day at two nearby locations (within 50 km) off Peru, thus examining temporal variations in habitat and resource use through their life span. Our objective was to look at intra- and inter-individual isotopic variations to investigate the overall isotopic niche of jumbo squids and their degree of specialization. As this species is thought to be highly migratory [14] and to have ontogenic shifts in its diet [13], [18], we expected strong variations in its isotopic carbon and nitrogen gladius profiles. Finally, variability in food habits of large jumbo squids was also investigated by the traditional method of stomach content analyses of several individuals sampled along the northern coast of Peru. These stomach analyses represent a snapshot in animal's diet and contrast with life history reconstructions allowed by longitudinal investigations along the gladius, but they nicely complement our isotopic analyses.

## Materials and Methods

### Ethics statement

Animals in this study were cared for in accordance with the guidelines of the ethics committee of the Institute of Research for Development and the Sea Institute of Peru (agreement 006481/01).

### Squid sampling

**Figure 1:**
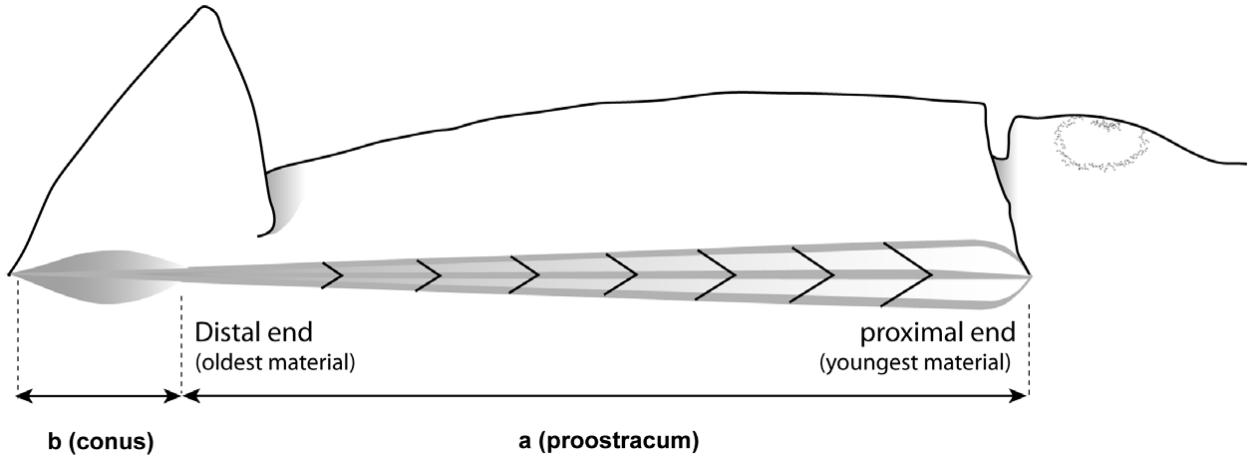
Jumbo squid gladius and isotopic sampling. Schematic dorsal view of *Dosidicus gigas* gladius (adapted from Perez et al. 1996). Sampling was done through 1 cm sections along the gladius proostracum (a) without sampling the conus (b) following the V-shape of the growth lines. Sections close to the proximal end correspond to the most recent formed material and to the oldest age of squid.

Gladii and statoliths of large (>60 cm ML) jumbo squids were sampled from 5 adults collected in offshore waters of Northern Peru on November 19, 2008 (Table 1). They were stored in ethanol and then frozen at −20°C. Age was estimated by counting the number of increments per statolith following [19]. Gladii were cleaned in distilled water for 5 min in an ultrasonic bath. They were then measured, dried and cut into 1 cm consecutive sections respecting the V shape of the growth lines following [13], Fig. 1. The sections sampled at the proximal end of the gladius (near the head) correspond to the most recently deposited material produced when the animal is an adult (oldest age), while distal sections of the gladius correspond to older material deposited when the animal was young. Only the proostracum was sampled, resulting in 50 to 62 sections per individual for gladii ranging between 65 and 80 cm length. Each cm of gladius averages different amounts of time (time-averaging, see 20) because of the 3-dimensional nature of gladius growth and varying growth rates along squids' life [20], [21]. Consequently, proximal (youngest) cm-wide sections represent shorter time periods than distal (oldest) sections. Sections (n = 274) were ground into powder, weighed with a micro-balance, and packed in tin containers for isotopic analysis. The main constituents of gladius are chitin and proteins [22], [23]. Hence, with chitin being depleted in ^15^N compared to proteins and diet, muscle isotope values of the corresponding individuals were also analyzed to control for these differences. Muscles were dried, ground to a fine powder and lipids were removed using cyclohexane [24]. Finally, the gladius and lipid-free muscle of six small (4–5 cm ML) jumbo squid individuals sampled along the coast of Peru in February 2009 at 3 and 9°S were analyzed to track latitudinal variations in baseline stable isotope values. Their gladius was cut into 1 cm consecutive sections following the method used for larger squids.

**Table 1:** Age, size, coordinates, isotope values and C/N mass ratios of the five large jumbo squids.

			Muscle				Gladius											
			δ^15^N	δ^13^C	C/N		δ^15^N				δ^13^C				C/N			
Size (cm)	Coordinates	Age (days)				N	Mean	Max	Min	Range	Mean	Max	Min	Range	Mean	Max	Min	Range
**65.2**	8°37′S, 80°42′W	**258**	**12.5**	**−15.9**	**3.2**	**50**	**8.2**	**9.2**	**7.5**	**0.7**	**−15.8**	**−14.9**	**−16.6**	**1.7**	**3.9**	**4.1**	**3.8**	**0.3**
72.2		ND	14.8	−16.0	3.2	53	10.0	12.5	7.4	2.7	−16.1	−15.0	−17.0	2.0	3.8	4.0	3.8	0.2
71.2		242	11.5	−16.5	3.2	58	6.9	7.5	6.4	0.5	−16.9	−16.0	−17.6	1.7	3.8	4.0	3.7	0.3
**81.2**	8°14′S, 80°32′W	**276**	**17.7**	**−16.2**	**3.2**	**62**	**12.5**	**14.6**	**9.6**	**2.9**	**−16.4**	**−15.5**	**−16.8**	**1.3**	**3.8**	**4.0**	**3.8**	**0.2**
66.5		252	12.4	−15.6	3.2	51	7.4	8.4	6.5	0.9	−15.7	−14.9	−16.7	1.8	3.9	4.0	3.8	0.2

**Figure 2:**
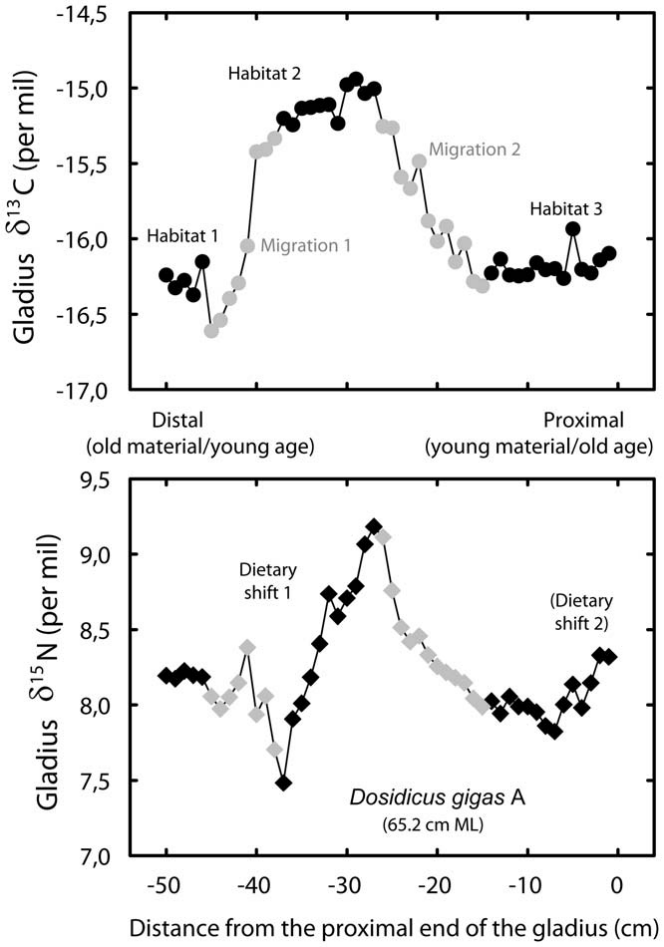
High resolution isotopic profile along individual A gladius. δ^13^C (filled circles) and δ^15^N (filled diamonds) values along the length of the gladius of a 65.2 cm ML jumbo squid. Grey symbols represent periods of migration, while black symbols illustrate a more fixed isotopic habitat.

**Figure 3:**
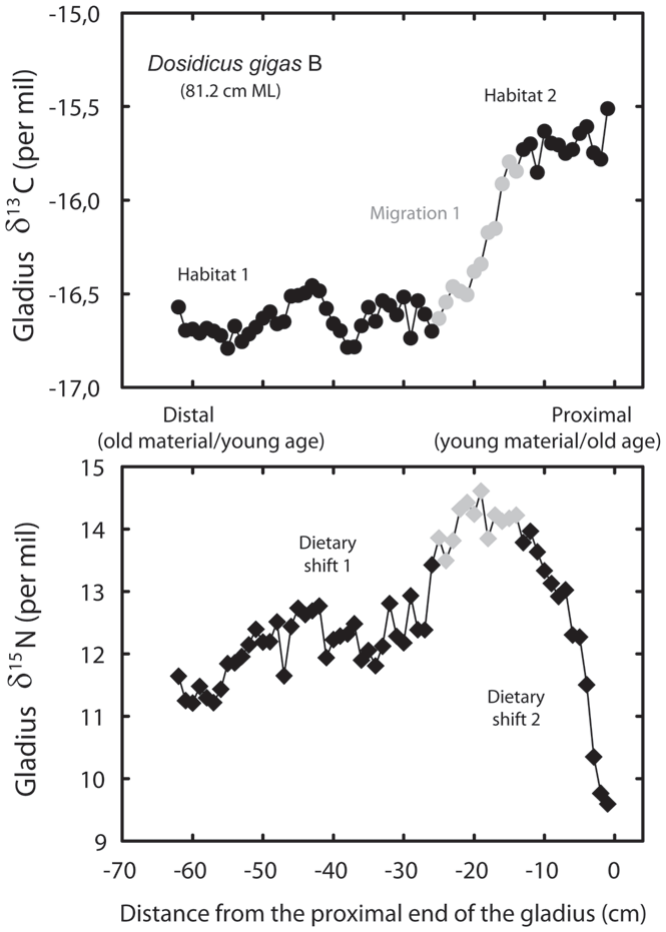
High resolution isotopic profile along individual B gladius. δ^13^C (filled circles) and δ^15^N (filled diamonds) values along the length of the gladius of a 81.2 cm ML jumbo squid. Grey symbols represent period of migration, while black symbols illustrate a more fixed isotopic habitat.

Bold values correspond to individuals represented on figs 2 and 3. N corresponds to the number of serial samples along the gladius.

### Isotopic analysis

Samples were analyzed using an elemental analyzer (Flash EA 1112, Thermo Scientific, Milan, Italy) coupled to an isotope ratio mass spectrometer (Delta V Advantage with a Conflo IV interface, Thermo Scientific, Bremen, Germany). Results are expressed in standard δ notation based on international standards (Vienna Pee Dee Belemnite for δ^13^C and N_2_ in air for δ^15^ N) following the formula: δ^13^C or δ^15^N = [(*R*_sample/_ *R*_standard_)−1]×10^3^ (in ‰), where *R* is ^13^C/^12^C or ^15^N/^14^N. Reference gas calibration was done using reference materials (USGS-24, IAEA-CH6, IAEA-600 for carbon; IAEA-N1, -N2, -N3, -600 for nitrogen). Analytical precision based on isotope values of the acetanilide (Thermo Scientific) used to estimate C and N content for each sample series was <0.1‰ both for carbon and nitrogen. Values are means ± s.d.

### Stomach content analysis

Stomach were collected from 37 large jumbo squids (range 65–85 cm ML) caught during 2008 along a latitudinal gradient (from 4°S to 11°S). They were kept frozen (−20°C) until analysis. In the laboratory, fresh remains were divided into the main prey items, which were weighed to calculate their proportion by mass in the diet. Prey items were identified to the minimum possible taxon using published keys and descriptions and by comparison with material held in our own reference collection, using fish otoliths and bones, cephalopod beaks and crustacean exoskeletons.

## Results

Assuming a deposition rate of one increment per day in statoliths, the lifespan of the five large individuals was ∼8–9 months (257±14 days), and ∼2 months (57±7 days) for the six small individuals (4–5 cm ML) (Table 1, 2). C/N ratios were consistent among and within individuals (range: 3.8–4.1), thus indicating similar biochemical composition of the samples allowing inter- and intra-individual comparisons (Table 1). Gladii isotopic signatures spread over a relatively small range of δ^13^C values (from −17.6 to −14.9‰, a 2.7‰ difference), but over a much larger range of δ^15^N values (from 6.4 to 14.6‰, a 8.2‰ difference). The same patterns were observed with muscle isotopic values, with δ^13^C ranging from −16.5 to −15.6‰ and δ^15^N from 11.5 to 17.7‰ (Table 1). Mean muscle and gladius isotope values were highly correlated for both δ^13^C (δ^13^C_muscle_ = 0.67×δ^13^C_gladius_−5.22, R^2^ = 0.94, n = 5) and δ^15^N (δ^15^N_muscle_ = 1.09×δ^15^N_gladius_+3.96, R^2^ = 0.99, n = 5). All data are provided in Table S1. Overall, the isotopic profiles along the gladius showed no consistency (i.e. they had different trajectories) both between and within the five large individuals, with all isotopic records shifting through time (Fig. S1).

**Table 2:** Age, size, isotopic values (± s.d) and C/N mass ratios of three small individuals per latitude.

			Muscle				Gladius											
			δ^15^N	δ^13^C	C/N		δ^15^N				δ^13^C				C/N			
Size (cm)	Coordinates	Age (days)				N	Mean	Max	Min	Range	Mean	Max	Min	Range	Mean	Max	Min	Range
6.4	3°29′S, 81°59′W	68	9.5	−18.9	3.3	4	5.1	5.9	4.3	1.6	−19.2	−19,0	−19.4	0.4	4.1	4.2	3.9	0.3
5.2		66	9.6	−19.0	3.3	3	4.9	5.1	4.7	0.4	−19.3	−19.2	−19.4	0.2	4.3	4.3	3.9	0.4
4.1		56	9.1	−18.8	3.3	3	4.8	5.2	4.5	0.7	−19.3	−19.2	−19.6	0.6	4.0	4.2	3.9	0.3
4.2	9°12′S, 79°10′W	56	14.5	−16.3	3.3	3	10.5	11.0	10.0	1.0	−16.3	−16.2	−16.4	0.2	4.0	4.0	4.0	0.0
4,0		53	14.6	−16.3	3.3	3	10.1	11.0	9.6	1.4	−16.2	−16,0	−16.4	0.4	4.0	4.0	3.9	0.1
4.4		52	14.7	−16.2	3.2	3	9.8	10.0	9.3	0.7	−16.3	−16,0	−16.6	0.6	4.1	4.2	4.1	0.1

N corresponds to the number of serial samples along the gladius.

In all squids, intra-individual gladius δ^13^C variations were substantial (from 1.3 to 2.0‰), indicating foraging along isotopic gradients. Nitrogen isotopes also showed temporal variations through time (from 1.1 to 5.0‰) with no systematic increase through size/age. Two of the five individuals (individuals A and B) adopted contrasting foraging strategies (Figs. 2, 3), i.e. they showed similar δ^13^C values, but their mean δ^15^N values (8.2 vs. 12.5‰) and ranges were different (Table 1). This strong isotope difference in gladius δ^15^N values among individuals A and B was also observed in their muscle isotopic values (12.5 vs. 17.7‰). Noticeably, individual B showed a strong δ^15^N decrease (4.6‰) at the end of its life (just before capture).

Isotopic values of small squid gladii (4–5 cm) sampled at 3°S and 9°S (Table 2) revealed strong latitudinal differences in isotopic baseline levels for both δ^13^C (3.4‰ range) and δ^15^N values (5.2‰ range). Indeed, trophic level differences alone cannot account for these large variations in such small animals. As for large squids, mean muscle and gladius isotope values were positively correlated (n = 6, δ^13^C_muscle_ = 0.88×δ^13^C_gladius_−1.14, R^2^ = 1.00; δ^15^N_muscle_ = 0.99×δ^15^N_gladius_+4.52, R^2^ = 0.99).

Overall, stomach content analysis across northern Peru revealed that large jumbo squids feed on a large diversity of prey (Table 3). Food was dominated by fish (in particular *Vinciguerria* sp., Nomeidae) and cephalopods (*Dosidicus gigas* and other species) but crustaceans such as euphausiids can also make a large contribution to the diet (up to 45% by mass).

**Table 3:** Diet (% by mass) of large jumbo squids (65–85 cm ML) collected off Peru (4–11°S) during 2008.

	Latitude		
	4–5°S	8–9°S	10–11°S
**Prey**			
*Dosidicus gigas*	**53.8**	6.2	
Other Cephalopods		**62.2**	0.1
Euphausiids	**45.4**		
*Pleuroncodes monodon*	0.1		
Myctophidae	0.3		0.1
*Vinciguerria* sp.		**30.6**	2.1
Nomeidae			**96.6**
Other Fishes	0.4	1.0	1.1
**Other information**			
Number of stomachs	14	16	7
% full stomachs	85.7	62.5	85.7

## Discussion

We report here the first results on jumbo squid trophic isotope ecology off Peru using sequential stable isotope values along the gladius of five large individuals. Previous work [13] reported that the jumbo squid gladius is a powerful tool to determine the geographic origin of squids and their ontogenic variations, but they did not investigate variations within and between individuals at a given location. Our results reveal strong intra- and inter-individual variations in stable isotope values along gladii of jumbo squids, underlining the potential of the isotopic tool to depict individual variations in foraging strategies.

In marine ecosystems, δ^13^C values vary greatly with latitude and/or inshore/offshore gradients [10], [25]. Carbon isotope values of small individuals caught at 3 and 9°S showed a 3‰ δ^13^C variation, confirming the presence of latitudinal gradients of the isotopic baseline along the coast of Peru. We cannot however discriminate between this latitudinal and a potential neritic/oceanic gradient in our δ^13^C values. The δ^13^C values of the most recent gladius sections of the five large individuals captured on the same day (B, C and A, D, E) were similar (−15.5, −15.7 and −16.1, −16.4, −16.3‰, respectively), suggesting foraging within the same habitat before capture. Intra-individual δ^13^C variations on gladius isotopic profiles (Figs. 2, 3) were interpreted as reflecting either migrations or more resident periods. Flat lying (invariant) sections of the carbon isotope profiles are assumed to represent resident locations or spatially limited movements (i.e., habitat 1, 2, 3 on Figs. 2, 3), while sections where the δ^13^C profiles vary (grey symbols) represent a migration between habitats. With resident and migration period defined, intra-individual δ^15^N variations during resident periods (i.e., within habitats 1, 2 or 3; Figs. 2, 3) was interpreted as a change in trophic position. Variability in δ^15^N profiles during migrations (changing δ^13^C), however, could not be interpreted since baseline δ^15^N variations may occur during squid migrations. Indeed, the δ^15^N values of the six small squids sampled from 3°S to 9°S off Peru suggested such baseline isotopic changes with a 5.2‰ δ^15^N variation along a 6° latitudinal gradient. The eastern South Pacific off Peru encompasses one of the most intense and shallow oxygen minimum zones (OMZ) of the world's oceans [26], [27], [28] and these OMZs are generally the site of intense denitrification [29] that is known to increase the baseline δ^15^N values [30], [31]. In such productive regions, denitrification preferentially removes ^15^N-depleted NO_3-_ and leaves residual nitrate ^15^N-enriched [32]. Variable anoxic conditions along the Peruvian coast [e.g., strong anoxic conditions occurring south of 5–10°S, 28] can therefore contribute to latitudinal gradients in the δ^15^N baseline level that are reflected in organisms at higher trophic levels.

The observed intra-individual δ^13^C variations in jumbo squid gladii revealed a complex life history (i.e. several migrations). Noticeably, overlapping δ^13^C values among individuals were observed, but every squid is unique and exhibits a different isotopic pattern through its life. Using biologging, Bazzino et al. [33] showed that 4 large jumbo squids (>70 cm ML) displayed different horizontal movements within 2–4 weeks of life [33]. They proposed that these changes reflect alterations in foraging behaviour, and searches for optimal foraging locations [33]. Their findings of short time scale migrations [33] are consistent with the long-term differences in gladius isotopic variations we observed among individuals. However, the relatively small intra-individual variations in δ^13^C values together with overlapping values among the five individuals suggest that all individual sampled probably foraged in a restricted area at the scale of northern Peru. Furthermore, the observed intra and inter-individual differences never reached the 3‰ δ^13^C difference observed between small jumbo squids sampled at 3°S and 9°S.

Concerning δ^15^N variations, the profiles of the two individuals A and B illustrate the variability in foraging strategies (Figs. 2, 3). While the individual A showed two periods of dietary change that can be interpreted by increases in trophic levels, individual B showed a very different and distinct pattern. A 2.2‰ δ^15^N increase early in life was followed by a strong 4.6‰ drop, which illustrates a decrease in its trophic level (probably from fish to euphausiids) at the end of its life. Euphausiids have been found to make a large contribution to the diet of large jumbo squids off Chile [>38% in mass, 2]. Stomach contents collected off Peru also showed that they forage for a large variety of prey, including euphausiids (Table 3). Our results therefore reveal the high variability of trophic patterns of large jumbo squids, and refute the systematic increase of trophic level through ontogeny reported by most studies in squids [e.g. 8], [12], [13], [18], except by [34] who did not find ontogenic differences in jumbo squids (14–87 cm) diet in the Gulf of California.

Interestingly, muscle δ^15^N values of individuals A and B (12.5 vs. 17.7‰) would have lead us to incorrectly conclude that they had different trophic levels, with squid B probably being cannibalistic. However, gladius analyses showed that δ^15^N values were different throughout almost all their life, suggesting that the two individuals probably came from different locations with different isotopic baselines. Individual B, which presented a much higher δ^15^N, most probably came from further south where the OMZ is more intense (and therefore so is denitrification) and shallower [28], [35], both which would increase the δ^15^N values of the baseline level (as is suggested by the highest δ^15^N values of the 3 small jumbo squids sampled at 9°S, Table 2). Furthermore, the strong decrease of trophic level at the end of the life of individual B seems to preclude a cannibalistic comportment and even to suggest a lower trophic level than individual A.

Finally, simultaneous analysis of gladius and muscle in both small and large squids showed a strong correlation between average muscle and gladius isotopic values, with similar values for δ^13^C (0.2±0.2‰ difference) but consistently lower δ^15^N values for gladius (−4.5±0.3‰) compared to muscles. It must be kept in mind however that gladius and muscles have different metabolic turnover rates. Muscle values correspond to an integration of a few weeks of life whereas average values provided for gladius correspond to the squid's entire life. The correlations and average differences between isotopic values of muscles and gladius are therefore only valid for the small individuals (2 months old) where muscle and gladius match the same time period. These consistently lower δ^15^N values in gladius are due to the presence of chitin, being depleted in ^15^N (but not in ^13^C) compared to proteins which are the main constituents of muscles [22], [23]. The knowledge of this 4.5‰ difference between gladii and muscles can be useful for comparison purposes in future studies where only one tissue is available, for example to calculate trophic positions through gladius samples.

In summary, these results show that jumbo squids living in the same environment at a given time can have completely different historical backgrounds. The data suggest that (i) jumbo squid actively migrate, (ii) they do not have a fixed diet and foraging strategy, and (iii) they do not systematically increase their trophic level with age. These differences in life history strategies confirm the high degree of plasticity of the species and its high potential to rapidly colonize new areas and adapt to environmental variability. The next step will be to use stable isotopes in combination with tagging studies to better assess the time-changes in habitats and their associated resources that characterize the life history of jumbo squids. Stable isotopes in squid gladii therefore offer an excellent opportunity to depict high resolution and ontogenic variations in individual foraging strategies of squids, resolving some bias inherent to stomach content analysis and stable isotopes on metabolically active tissues.

## Supporting Information

Figure S1 **δ^13^C (upper panel) and δ^15^N (lower panel) values along the length of the gladius of the five large jumbo squids. Grey and black symbols represent sampling at different places.**
(TIF)

Table S1 **Gladius isotopic values and C/N mass ratios of individuals A to E.**
(DOC)

## References

[1] Rodhouse PG, Nigmatullin CM (1996). Role as consumers.. Phil Trans R Soc B.

[2] Ibañez CM, Arancibia H, Cubillos LA (2008). Biases in determining the diet of jumbo squid *Dosidicus gigas* (D'Orbigny 1835) (Cephalopoda: Ommastrephidae) off southern-central Chile (34°S–40°S).. Helgol Mar Res.

[3] Bolnick DI, Svanback R, Fordyce JA, Yang LH, Davis JM (2003). The ecology of individuals: incidence and implications of individual specialization.. Am Nat.

[4] Bearhop S, Adams CE, Waldron S, Fuller RA, Macleod H (2004). Determining trophic niche width: a novel approach using stable isotope analysis.. J Anim Ecol.

[5] Cherel Y, Kernaléguen L, Richard P, Guinet G (2009). Whisker isotopic signature depicts migration patterns and multi-year intra- and inter-individual foraging strategies in fur seals.. Biol Lett.

[6] Newsome SD, Tinker MT, Monson DH, Oftedal OT, Ralls K (2009). Using stable isotopes to investigate individual diet specialization in California sea otters (*Enhydra lutris nereis*).. Ecology.

[7] Vander Zanden HB, Bjorndal KA, Reich KJ, Bolten AB (2010). Individual specialists in a generalist population: results from a long-term stable isotope series.. Biol Lett.

[8] Guerra A, Rodriguez-Navarro AB, Gonzalez AF, Romanek CS, Alvarez-Lloret P (2010). Life-history traits of the giant squid *Architeuthis dux* revealed from stable isotope signatures recorded in beaks.. ICES J Mar Sci.

[9] Vanderklift MA, Ponsard S (2003). Source of variation in consumer- diet δ^15^N enrichment: a meta-analysis.. Oecologia.

[10] Cherel Y, Hobson KA (2007). Geographical variation in carbon stable isotope signatures of marine predators: a tool to investigate their foraging areas in the Southern Ocean.. Mar Ecol Prog Ser.

[11] Jaeger A, Lecomte VJ, Weimerskirch H, Richard P, Cherel Y (2010). Seabird satellite tracking validates the use of latitudinal isoscapes to depict predators' foraging areas in the Southern Ocean.. Rapid Commun Mass Spectrom.

[12] Cherel Y, Fontaine C, Jackson GD, Jackson CH, Richard P (2009). Tissue, ontogenic and sex-related differences in δ^13^C and δ^15^N values of the oceanic squid *Todarodes filippovae* (Cephalopoda: Ommastrephidae).. Mar Biol.

[13] Ruiz-Cooley RI, Villa EC, Gould WR (2010). Ontogenetic variation of δ^13^C and δ^15^N recorded in the gladius of the jumbo squid *Dosidicus gigas*: geographic differences.. Mar Ecol Progr Ser.

[14] Nigmatullin CHM, Nesis KN, Arkhipkin AI (2001). A review of the biology of the jumbo squid *Dosidicus gigas* (Cephalopoda:Ommastrephidae).. Fish Res.

[15] Bograd SJ, Castro CG, Di Lorenzo E, Palacios DM, Bailey G (2008). Oxygen declines and the shoaling of the hypoxic boundary in the California Current.. Geophys Res Lett.

[16] Zeidberg LD, Robison BH (2008). Invasive range expansion by the Humboldt squid, *Dosidicus gigas*, in the eastern.. North Pacific Proc Natl Acad Sci.

[17] Argüelles J, Tafur R, Taipe A, Villegas P, Keyl F (2008). Size increment of jumbo flying squid *Dosidicus gigas* mature females in Peruvian waters, 1989–2004.. Prog Oceanogr.

[18] Ruiz-Cooley RI, Markaida U, Gendron D, Aguiñiga S (2006). Stable isotopes in jumbo squid (*Dosidicus gigas*) beaks to estimate its trophic position: comparison between stomach content and stable isotopes.. J Mar Biol Ass of the UK.

[19] Arkhipkin AI, Jereb P, Ragonese S, Boletzky SV (1991). Methods for cephalopod age and growth studies with emphasis on statolith ageing techniques..

[20] Goodwin DH, Schöne BR, Dettman DL (2003). Resolution and _delity of oxygen isotopes as paleotemperature proxies in bivalve mollusk shells: Models and observations.. Palaios.

[21] Perez JAA, O'Dor RK, Beck P, Dawe EG (1996). Evaluation of gladius dorsal surface structure for age and growth studies of the short-finned squid, *Illex illecebrosus* (Teuthoidea:Ommastrephidae).. Can J Fish Aquat Sci.

[22] DeNiro MJ, Epstein S (1978). Influence of diet on the distribution of carbon isotopes in animals.. Geochim Cosmochim Acta.

[23] Webb S, Hedges REM, Simpson SJ (1998). Diet quality influences the δ^13^C and δ^15^N of locusts and their biochemical components.. J Exp Biol.

[24] Kojidanovik J, Richard P, Le Corre M, Cosson RP, Bustamante P (2008). Effects of lipid extraction on δ^13^C and δ^15^N values in seabird muscle, liver and feathers.. Waterbirds.

[25] Rau GH, Sweeney RE, Kaplan IR (1982). Plankton ^13^C:^12^C ratio changes with latitude: differences between northern and southern oceans.. Deep-Sea Res I.

[26] Chavez F, Bertrand A, Guevara-Carrasco R, Soler P, Csirke J (2008). The northern Humboldt Current System: Brief history, present status and a view towards the future.. Prog Oceanogr.

[27] Paulmier A, Ruiz-Pino D (2009). Oxygen minimum zones (OMZs) in the modern ocean.. Prog Oceanogr.

[28] Bertrand A, Ballón M, Chaigneau A (2010). Acoustic observation of living organisms reveals the oxygen minimum zone.. PLoS ONE.

[29] Naqvi SWA, Jayakumar DA, Narvekar PV, Naik H, Sarma VVSS (2000). Increased marine production of N2O due to intensifying anoxia on the Indian continental shelf.. Nature.

[30] Sigman DM, Altabet MA, McCorkle DC, Francois R, Fischer G (1999). The δ^15^N of nitrate in the Southern Ocean: nitrate consumption in surface waters.. Global Biogeochem Cycles.

[31] Graham BS, Koch PL, Newsome SD, McMahon KW, Aurioles D, West JB, Bowen GJ, Dawson TE, Tu KP (2010). Using isoscapes to trace movements and foraging behaviour of top predators in oceanic ecosystems.. Isoscapes: Understanding Movement, Pattern, and Process on Earth through Isotope Mapping.

[32] Voss M, Dippner JW, Montoya JP (2001). Nitrogen isotope patterns in the oxygen-deficient waters of the Eastern Tropical North Pacific Ocean.. Deep-Sea Res.

[33] Bazzino G, Gilly WF, Markaida U, Salinas-Zavala CA, Ramos-Castillejos R (2010). Horizontal movements, vertical-habitat utilization and diet of the jumbo squid (*Dosidicus gigas*) in the Pacific Ocean off Baja California Sur, Mexico.. Progr Oceanogr.

[34] Markaida U, Sosa-Nishizaki O (2003). Food and feeding habits of jumbo squid *Dosidicus gigas* (Cephalopoda: Ommastrephidae) from the Gulf of California, Mexico.. J Mar Biol Assoc UK.

[35] Fuenzalida R, Schneider W, Garcés-Vargas J, Bravo L, Lange C (2009). Vertical and horizontal extension of the oxygen minimum zone in the eastern South Pacific Ocean.. Deep Sea Res.

