# Supplementary figures and images for: Sequential Isotopic Signature Along Gladius Highlights Contrasted Individual Foraging Strategies of Jumbo Squid (Dosidicus gigas)
Source: PLoS One. 2011 Jul 14;6(7):e22194. doi: 10.1371/journal.pone.0022194 (PMC3136502; doi:10.1371/journal.pone.0022194)

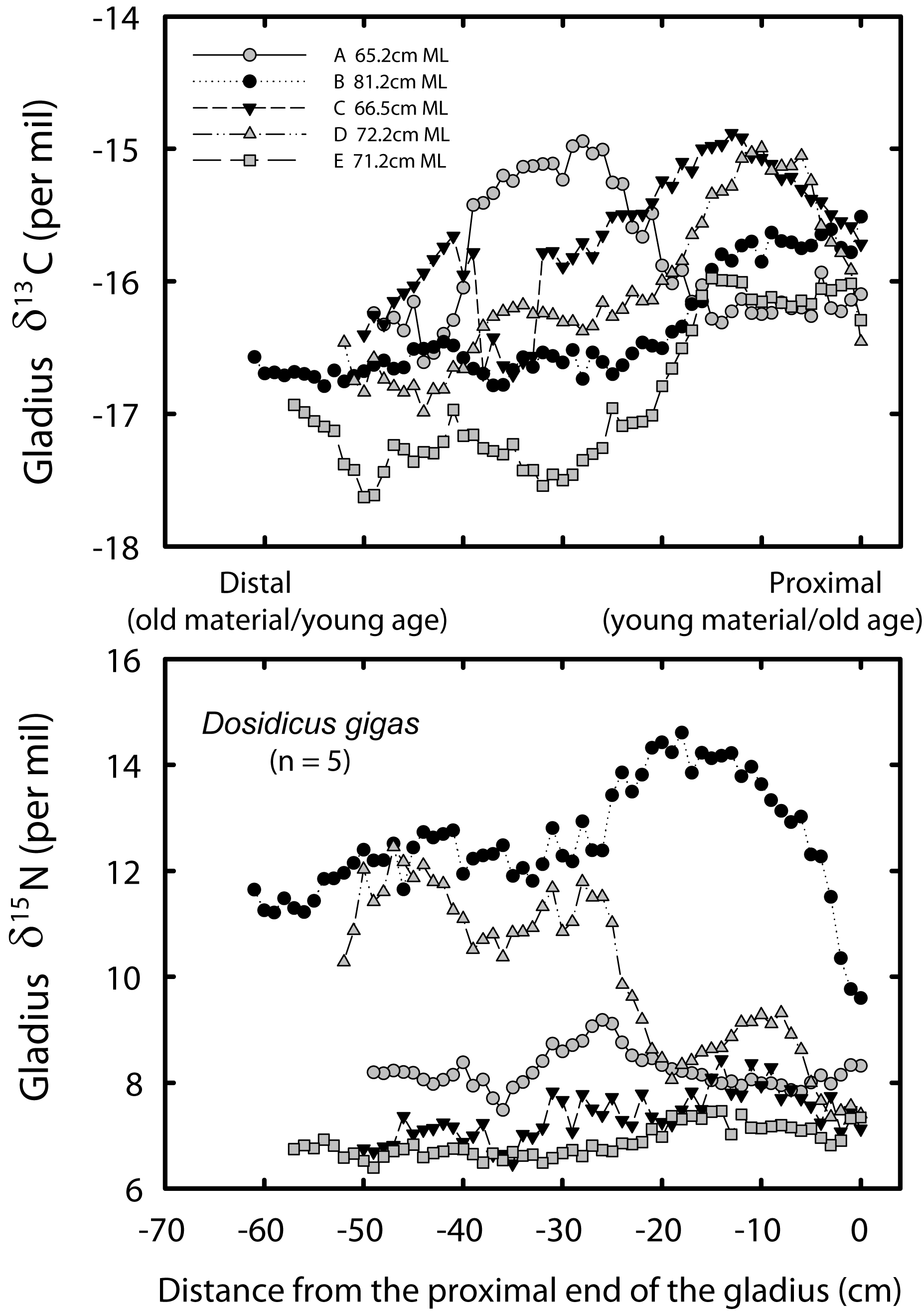

Supplement: Figure S1 — δ13C (upper panel) and δ15N (lower panel) values along the length of the gladius of the five large jumbo squids. Grey and black symbols represent sampling at different places. (TIF) [file pone.0022194.s001.tif]
