# Supplementary material for: Sequential Isotopic Signature Along Gladius Highlights Contrasted Individual Foraging Strategies of Jumbo Squid (Dosidicus gigas)
Source: PLoS One. 2011 Jul 14;6(7):e22194. doi: 10.1371/journal.pone.0022194 (PMC3136502; doi:10.1371/journal.pone.0022194)
Supplement: Table S1 — Gladius isotopic values and C/N mass ratios of individuals A to E. (DOC) [file pone.0022194.s002.doc]

Table S1. Gladius isotopic values and C/N mass ratios of individuals A to E.

| Ind. A: 65.2 cm ML | n° sample | δ^13^C | δ^15^N | C/N |
| --- | --- | --- | --- | --- |
| proximal (recent material) | 1 | -16,10 | 8,32 | 3,92 |
|  | 2 | -16,14 | 8,33 | 3,93 |
|  | 3 | -16,23 | 8,15 | 3,91 |
|  | 4 | -16,20 | 7,98 | 3,91 |
|  | 5 | -15,94 | 8,14 | 3,93 |
|  | 6 | -16,26 | 8,00 | 3,94 |
|  | 7 | -16,20 | 7,82 | 4,03 |
|  | 8 | -16,21 | 7,86 | 3,94 |
|  | 9 | -16,16 | 7,95 | 3,95 |
|  | 10 | -16,24 | 7,99 | 3,92 |
|  | 11 | -16,25 | 7,99 | 3,94 |
|  | 12 | -16,24 | 8,06 | 4,04 |
|  | 13 | -16,14 | 7,94 | 3,94 |
|  | 14 | -16,23 | 8,03 | 3,97 |
|  | 15 | -16,31 | 7,99 | 4,03 |
|  | 16 | -16,28 | 8,04 | 4,05 |
|  | 17 | -16,03 | 8,15 | 3,93 |
|  | 18 | -16,15 | 8,18 | 3,87 |
|  | 19 | -15,92 | 8,22 | 3,89 |
|  | 20 | -16,02 | 8,26 | 3,98 |
|  | 21 | -15,88 | 8,33 | 3,92 |
|  | 22 | -15,49 | 8,46 | 3,91 |
|  | 23 | -15,67 | 8,42 | 3,90 |
|  | 24 | -15,59 | 8,51 | 3,90 |
|  | 25 | -15,27 | 8,76 | 3,95 |
|  | 26 | -15,26 | 9,11 | 3,95 |
|  | 27 | -15,01 | 9,18 | 3,88 |
|  | 28 | -15,04 | 9,07 | 3,90 |
|  | 29 | -14,94 | 8,79 | 3,89 |
|  | 30 | -14,98 | 8,71 | 3,85 |
|  | 31 | -15,24 | 8,59 | 3,91 |
|  | 32 | -15,11 | 8,74 | 3,86 |
|  | 33 | -15,12 | 8,41 | 3,86 |
|  | 34 | -15,13 | 8,18 | 3,84 |
|  | 35 | -15,14 | 8,01 | 3,85 |
|  | 36 | -15,24 | 7,91 | 3,83 |
|  | 37 | -15,20 | 7,48 | 3,84 |
|  | 38 | -15,34 | 7,70 | 3,82 |
|  | 39 | -15,41 | 8,06 | 3,84 |
|  | 40 | -15,42 | 7,94 | 3,82 |
|  | 41 | -16,05 | 8,38 | 3,90 |
|  | 42 | -16,29 | 8,15 | 3,92 |
|  | 43 | -16,40 | 8,05 | 3,89 |
|  | 44 | -16,54 | 7,97 | 3,90 |
|  | 45 | -16,61 | 8,06 | 3,89 |
|  | 46 | -16,15 | 8,19 | 3,86 |
|  | 47 | -16,37 | 8,20 | 3,87 |
|  | 48 | -16,28 | 8,23 | 3,87 |
|  | 49 | -16,33 | 8,17 | 3,88 |
| distal (old material) | 50 | -16,24 | 8,19 | 3,87 |
| Ind B: 81.2 cm ML | 1 | -15,51 | 9,60 | 3,95 |
| proximal (recent material) | 2 | -15,78 | 9,77 | 3,89 |
|  | 3 | -15,75 | 10,35 | 3,90 |
|  | 4 | -15,61 | 11,51 | 3,89 |
|  | 5 | -15,65 | 12,27 | 3,87 |
|  | 6 | -15,73 | 12,30 | 3,82 |
|  | 7 | -15,75 | 13,02 | 3,84 |
|  | 8 | -15,71 | 12,92 | 3,85 |
|  | 9 | -15,70 | 13,13 | 3,84 |
|  | 10 | -15,63 | 13,33 | 3,86 |
|  | 11 | -15,85 | 13,63 | 3,84 |
|  | 12 | -15,70 | 13,96 | 3,86 |
|  | 13 | -15,73 | 13,78 | 3,82 |
|  | 14 | -15,85 | 14,22 | 3,86 |
|  | 15 | -15,80 | 14,17 | 3,85 |
|  | 16 | -15,91 | 14,13 | 3,84 |
|  | 17 | -16,15 | 14,23 | 3,84 |
|  | 18 | -16,17 | 13,85 | 3,86 |
|  | 19 | -16,34 | 14,61 | 3,83 |
|  | 20 | -16,38 | 14,24 | 3,83 |
|  | 21 | -16,51 | 14,43 | 3,82 |
|  | 22 | -16,49 | 14,32 | 3,79 |
|  | 23 | -16,46 | 13,81 | 3,91 |
|  | 24 | -16,55 | 13,49 | 3,83 |
|  | 25 | -16,63 | 13,86 | 3,85 |
|  | 26 | -16,70 | 13,42 | 3,86 |
|  | 27 | -16,61 | 12,38 | 3,87 |
|  | 28 | -16,54 | 12,39 | 3,87 |
|  | 29 | -16,74 | 12,93 | 3,84 |
|  | 30 | -16,52 | 12,18 | 3,89 |
|  | 31 | -16,61 | 12,28 | 3,88 |
|  | 32 | -16,56 | 12,81 | 3,89 |
|  | 33 | -16,54 | 12,12 | 3,88 |
|  | 34 | -16,65 | 11,81 | 3,84 |
|  | 35 | -16,57 | 12,05 | 3,88 |
|  | 36 | -16,67 | 11,90 | 3,88 |
|  | 37 | -16,78 | 12,48 | 3,86 |
|  | 38 | -16,79 | 12,32 | 3,87 |
|  | 39 | -16,70 | 12,29 | 3,89 |
|  | 40 | -16,66 | 12,23 | 3,91 |
|  | 41 | -16,58 | 11,94 | 3,88 |
|  | 42 | -16,49 | 12,77 | 3,87 |
|  | 43 | -16,46 | 12,69 | 3,85 |
|  | 44 | -16,50 | 12,63 | 3,85 |
|  | 45 | -16,51 | 12,73 | 3,87 |
|  | 46 | -16,51 | 12,44 | 3,76 |
|  | 47 | -16,65 | 11,65 | 3,83 |
|  | 48 | -16,66 | 12,51 | 3,83 |
|  | 49 | -16,60 | 12,20 | 3,85 |
|  | 50 | -16,63 | 12,19 | 3,82 |
|  | 51 | -16,68 | 12,40 | 3,81 |
|  | 52 | -16,72 | 12,15 | 3,80 |
|  | 53 | -16,76 | 11,96 | 3,78 |
|  | 54 | -16,67 | 11,86 | 3,81 |
|  | 55 | -16,79 | 11,85 | 3,78 |
|  | 56 | -16,72 | 11,43 | 3,82 |
|  | 57 | -16,70 | 11,22 | 3,82 |
|  | 58 | -16,69 | 11,30 | 3,82 |
|  | 59 | -16,71 | 11,48 | 3,82 |
|  | 60 | -16,69 | 11,21 | 3,82 |
|  | 61 | -16,70 | 11,25 | 3,84 |
| distal (old material) | 62 | -16,57 | 11,64 | 3,89 |
| Ind C: 66.5 cm ML | 1 | -15,72 | 7,13 | 3,88 |
| proximal (recent material) | 2 | -15,59 | 7,48 | 3,85 |
|  | 3 | -15,55 | 7,07 | 3,86 |
|  | 4 | -15,50 | 7,75 | 3,85 |
|  | 5 | -15,40 | 7,24 | 3,84 |
|  | 6 | -15,38 | 7,56 | 3,83 |
|  | 7 | -15,30 | 7,70 | 3,84 |
|  | 8 | -15,21 | 7,87 | 3,83 |
|  | 9 | -15,22 | 7,70 | 3,85 |
|  | 10 | -15,12 | 8,29 | 3,88 |
|  | 11 | -15,07 | 7,95 | 3,86 |
|  | 12 | -15,05 | 8,36 | 3,84 |
|  | 13 | -14,92 | 7,77 | 3,85 |
|  | 14 | -14,88 | 7,81 | 3,84 |
|  | 15 | -14,97 | 8,44 | 3,84 |
|  | 16 | -14,98 | 8,09 | 3,82 |
|  | 17 | -15,00 | 7,50 | 3,85 |
|  | 18 | -15,16 | 7,82 | 3,82 |
|  | 19 | -15,10 | 7,49 | 3,84 |
|  | 20 | -15,28 | 7,21 | 3,81 |
|  | 21 | -15,24 | 7,25 | 3,84 |
|  | 22 | -15,41 | 7,36 | 3,83 |
|  | 23 | -15,49 | 7,79 | 3,83 |
|  | 24 | -15,50 | 7,19 | 3,86 |
|  | 25 | -15,50 | 7,29 | 3,86 |
|  | 26 | -15,51 | 7,72 | 3,84 |
|  | 27 | -15,65 | 7,39 | 3,84 |
|  | 28 | -15,81 | 7,51 | 3,86 |
|  | 29 | -15,71 | 7,78 | 3,87 |
|  | 30 | -15,82 | 7,08 | 3,87 |
|  | 31 | -15,89 | 7,67 | 3,87 |
|  | 32 | -15,78 | 7,82 | 3,86 |
|  | 33 | -15,78 | 7,15 | 3,90 |
|  | 34 | -16,57 | 6,98 | 3,81 |
|  | 35 | -16,61 | 7,02 | 3,80 |
|  | 36 | -16,71 | 6,47 | 3,77 |
|  | 37 | -16,64 | 6,65 | 3,78 |
|  | 38 | -16,43 | 6,64 | 3,79 |
|  | 39 | -16,70 | 7,23 | 3,80 |
|  | 40 | -15,78 | 7,00 | 3,95 |
|  | 41 | -15,95 | 6,88 | 3,95 |
|  | 42 | -15,66 | 7,17 | 3,93 |
|  | 43 | -15,74 | 7,24 | 3,89 |
|  | 44 | -15,83 | 7,14 | 3,89 |
|  | 45 | -15,94 | 7,11 | 3,93 |
|  | 46 | -16,03 | 7,03 | 3,91 |
|  | 47 | -16,09 | 7,36 | 3,88 |
|  | 48 | -16,15 | 6,82 | 3,86 |
|  | 49 | -16,32 | 6,79 | 3,84 |
|  | 50 | -16,26 | 6,70 | 3,85 |
| distal (old material) | 51 | -16,40 | 6,76 | 3,81 |
| Ind D: 72.2 cm ML | 1 | -16,46 | 7,40 | 3,99 |
| proximal (recent material) | 2 | -15,92 | 7,56 | 3,80 |
|  | 3 | -15,79 | 7,46 | 3,80 |
|  | 4 | -15,71 | 7,35 | 3,78 |
|  | 5 | -15,58 | 7,65 | 3,76 |
|  | 6 | -15,24 | 8,00 | 3,76 |
|  | 7 | -15,05 | 8,62 | 3,78 |
|  | 8 | -15,13 | 8,91 | 3,80 |
|  | 9 | -15,13 | 9,31 | 3,81 |
|  | 10 | -15,17 | 9,11 | 3,84 |
|  | 11 | -14,99 | 9,28 | 3,86 |
|  | 12 | -15,03 | 9,14 | 3,90 |
|  | 13 | -15,07 | 9,14 | 3,84 |
|  | 14 | -15,28 | 8,86 | 3,89 |
|  | 15 | -15,32 | 8,65 | 3,89 |
|  | 16 | -15,35 | 8,63 | 3,90 |
|  | 17 | -15,56 | 8,57 | 3,91 |
|  | 18 | -15,65 | 8,41 | 3,84 |
|  | 19 | -15,85 | 8,34 | 3,89 |
|  | 20 | -15,94 | 8,06 | 3,84 |
|  | 21 | -16,00 | 8,45 | 3,87 |
|  | 22 | -16,15 | 8,62 | 3,83 |
|  | 23 | -16,15 | 9,19 | 3,83 |
|  | 24 | -16,09 | 9,62 | 3,82 |
|  | 25 | -16,21 | 9,85 | 3,84 |
|  | 26 | -16,27 | 11,02 | 3,84 |
|  | 27 | -16,16 | 11,51 | 3,85 |
|  | 28 | -16,34 | 11,50 | 3,85 |
|  | 29 | -16,38 | 11,79 | 3,84 |
|  | 30 | -16,31 | 11,03 | 3,87 |
|  | 31 | -16,31 | 10,85 | 3,88 |
|  | 32 | -16,26 | 11,67 | 3,84 |
|  | 33 | -16,24 | 11,32 | 3,87 |
|  | 34 | -16,25 | 10,92 | 3,88 |
|  | 35 | -16,18 | 10,84 | 3,87 |
|  | 36 | -16,20 | 10,83 | 3,87 |
|  | 37 | -16,23 | 10,37 | 3,90 |
|  | 38 | -16,27 | 10,80 | 3,88 |
|  | 39 | -16,34 | 10,69 | 3,90 |
|  | 40 | -16,51 | 10,51 | 3,87 |
|  | 41 | -16,66 | 11,09 | 3,83 |
|  | 42 | -16,65 | 11,26 | 3,85 |
|  | 43 | -16,82 | 11,75 | 3,88 |
|  | 44 | -16,82 | 11,79 | 3,85 |
|  | 45 | -16,99 | 12,11 | 3,84 |
|  | 46 | -16,79 | 11,86 | 3,80 |
|  | 47 | -16,84 | 12,17 | 3,82 |
|  | 48 | -16,80 | 12,45 | 3,81 |
|  | 49 | -16,74 | 11,60 | 3,82 |
|  | 50 | -16,58 | 11,42 | 3,78 |
|  | 51 | -16,84 | 12,02 | 3,77 |
|  | 52 | -16,75 | 10,87 | 3,78 |
| distal (old material) | 53 | -16,46 | 10,28 | 3,82 |
| Ind E: 71.2 cm ML | 1 | -16,29 | 7,34 | 3,90 |
| proximal (recent material) | 2 | -16,02 | 7,32 | 3,83 |
|  | 3 | -16,03 | 6,91 | 3,83 |
|  | 4 | -16,07 | 6,82 | 3,81 |
|  | 5 | -16,06 | 6,96 | 3,81 |
|  | 6 | -16,17 | 7,13 | 3,82 |
|  | 7 | -16,15 | 7,09 | 3,79 |
|  | 8 | -16,19 | 7,16 | 3,79 |
|  | 9 | -16,16 | 7,20 | 3,80 |
|  | 10 | -16,12 | 7,17 | 3,79 |
|  | 11 | -16,15 | 7,14 | 3,83 |
|  | 12 | -16,14 | 7,15 | 3,86 |
|  | 13 | -16,01 | 7,40 | 3,81 |
|  | 14 | -16,00 | 7,02 | 3,69 |
|  | 15 | -15,99 | 7,47 | 3,82 |
|  | 16 | -15,98 | 7,45 | 3,79 |
|  | 17 | -16,11 | 7,32 | 3,84 |
|  | 18 | -16,37 | 7,37 | 3,85 |
|  | 19 | -16,51 | 7,31 | 3,84 |
|  | 20 | -16,66 | 7,37 | 3,79 |
|  | 21 | -16,79 | 6,97 | 3,82 |
|  | 22 | -17,01 | 7,12 | 3,83 |
|  | 23 | -17,06 | 6,88 | 3,80 |
|  | 24 | -17,07 | 6,84 | 3,81 |
|  | 25 | -17,09 | 6,85 | 3,84 |
|  | 26 | -16,96 | 6,70 | 3,87 |
|  | 27 | -17,26 | 6,72 | 3,84 |
|  | 28 | -17,30 | 6,81 | 3,85 |
|  | 29 | -17,35 | 6,61 | 3,86 |
|  | 30 | -17,46 | 6,73 | 3,87 |
|  | 31 | -17,50 | 6,67 | 3,88 |
|  | 32 | -17,46 | 6,57 | 3,86 |
|  | 33 | -17,54 | 6,49 | 3,85 |
|  | 34 | -17,43 | 6,64 | 3,84 |
|  | 35 | -17,43 | 6,62 | 3,87 |
|  | 36 | -17,23 | 6,69 | 3,87 |
|  | 37 | -17,31 | 6,54 | 3,87 |
|  | 38 | -17,28 | 6,67 | 3,89 |
|  | 39 | -17,26 | 6,49 | 3,90 |
|  | 40 | -17,16 | 6,65 | 3,86 |
|  | 41 | -17,17 | 6,75 | 3,86 |
|  | 42 | -16,97 | 6,76 | 3,86 |
|  | 43 | -17,21 | 6,70 | 3,86 |
|  | 44 | -17,30 | 6,67 | 3,86 |
|  | 45 | -17,29 | 6,59 | 3,84 |
|  | 46 | -17,36 | 6,83 | 3,83 |
|  | 47 | -17,27 | 6,74 | 3,82 |
|  | 48 | -17,24 | 6,71 | 3,86 |
|  | 49 | -17,44 | 6,60 | 3,86 |
|  | 50 | -17,62 | 6,40 | 3,88 |
|  | 51 | -17,63 | 6,52 | 3,86 |
|  | 52 | -17,43 | 6,66 | 3,85 |
|  | 53 | -17,38 | 6,58 | 3,84 |
|  | 54 | -17,13 | 6,81 | 3,86 |
|  | 55 | -17,10 | 6,92 | 3,87 |
|  | 56 | -17,06 | 6,76 | 3,89 |
|  | 57 | -16,99 | 6,82 | 3,94 |
| distal (old material) | 58 | -16,93 | 6,75 | 4,02 |
